# Laser Welding of Titanium/Steel Bimetallic Sheets with In Situ Formation of Fe_x_(CoCrNiMn)Ti_y_ High-Entropy Alloys in Weld Metal

**DOI:** 10.3390/ma17030623

**Published:** 2024-01-27

**Authors:** Dejia Liu, Zhe Ma, Nianlong Xue, Weixiong Wang, Shanguo Han

**Affiliations:** 1 School of Materials Science and Engineering, East China Jiaotong University, Nanchang 330013, China; 13027247532@126.com (Z.M.); 19862176773@163.com (N.X.); wilsonwong0508@163.com (W.W.); 2 Yang Jiang China-Ukraine E. O. PATON Institute of Technology, Yangjiang 529532, China; 3 China-Ukraine Institute of Welding, Guangdong Academy of Sciences, Guangzhou 510651, China

**Keywords:** TA1/Q345 bimetallic sheets, high-entropy alloys, microstructure, mechanical properties

## Abstract

Due to the notable disparities in the physical and chemical characteristics between titanium and steel, the direct fusion of titanium/steel bimetallic sheets results in a considerable formation of fragile intermetallic compounds, making it difficult to achieve excellent metallurgical welded joints. In this study, a multi-principal powder of CoCrNiMn was designed and utilized as a filler material in the welding of the TA1/Q345 bimetallic sheet. It was expected that the in situ formation of Fe_x_(CoCrNiMn)Ti_y_ high-entropy alloys would be achieved using the filler powders, combined with the Ti and Fe elements from the melting of the TA1 and Q345 so as to restrain the generation of Fe-Ti IMCs and obtain the promising welded joints of the TA1/Q345 bimetallic sheet. An interesting finding is that high-entropy alloys were successfully obtained in the weld metal. The Fe-Ti intermetallic compounds at the welding interface were significantly reduced. The tensile strength was ~293 MPa, accounting for 60% of the strength of the base metal. Dimples were observed at the fracture of the welded joint.

## 1. Introduction

The cost-effectiveness of steel, along with the exceptional ability of titanium (Ti) to resist corrosion [1,2,3], renders Ti/steel bimetallic sheets highly suitable for diverse applications in industries such as aviation, petrochemicals, and marine environments [4]. In general, laser welding proves to be an effective method for dissimilar metal joining. However, due to the metallurgical incompatibility between Ti and steel, brittle intermetallic compounds (IMCs) are easily formed at the interface during direct fusion welding. This leads to difficulties in achieving a reliable welded joint of Ti/steel bimetallic sheets [5,6,7]. Relevant research shows that the brittle FeTi and Fe_2_Ti, which have high hardness values of ~600 HV and ~1000 HV, respectively [8], significantly reduce the mechanical properties of Ti/steel joints. Furthermore, there is a significant difference in thermal stress between Ti and steel during the welding process [9,10,11]. Consequently, it is easy to prompt the formation of cracks at the welding interface. The aforementioned issues are the primary challenges in achieving the laser welding of Ti/steel bimetallic sheets.

As of yet, many scholars have studied the dissimilar laser welding of Ti to steel. Tomashchuk et al. [12] have successfully achieved the bonding of Ti alloy and AISI 316L stainless steel through the method of laser offset. However, the welded joint exhibited a tensile strength of 350 MPa. Furthermore, Zhang et al. [7] meticulously explored the impact of different laser offset distances on the lap joints of Ti and steel. The results of the test indicated that, when the laser was positioned 0.5 mm apart from the welding point, the welded joint exhibited the highest strength of 263 MPa. A large amount of Ti-Cu IMCs were present at the weld metal (WM)/TA1 interfaces [7]. Zhu et al. [13] have investigated the properties and microstructure of Ti-steel welded joints under different heat inputs. A joint strength of ~420 MPa was achieved with a lower heat input. Those studies proved that laser welding can finish the dissimilar welding of Ti to steel. However, the mechanical properties of the welded joints have a close relationship with the laser welding parameters.

At present, the most widely employed method for the dissimilar welding of Ti to steel is to use filler materials that will not react with Fe or Ti to form intermediate layers. Cu [9,14], V [15], Nb [16], Mo [10], Ni [17], and their alloys were considered to be ideal intermediate materials for joining Ti to steel. Li et al. [18] investigated the gas tungsten arc welding of TC4 alloy to 304 stainless steel by utilizing CuSi_3_ wire as a filler material. The results found that a large amount of Fe_2_Ti was produced at the welding interface near the steel side. FeTi and a variety of Ti-Cu IMCs were generated at the welding interface close to Ti [18]. In addition, amorphous brazing materials were also widely used to deal with the challenges of the dissimilar joining of Ti and steel. By utilizing the Ti_35_Zr_25_Be_40−x_Co_x_ (x = 2, 4, 6, 8, 10) amorphous ribbon for brazing the TC4 alloy/316L stainless steel, a high shear strength of 225 MPa and an unavoidable reactive layer that was rich in Fe-Ti IMCs were reported [19]. Yao et al. [20] have examined the interfacial microstructure and mechanical properties of the welded joints using AgCuNi foil as the interlayer. Furthermore, for fusion welding, an AgCuTi filler metal was chosen to achieve the welding of 304 stainless steel and TC4 alloy [3]. The experiment demonstrated that, due to the atomic diffusion leading to the formation of Ti_2_Cu and AgTi_2_ at the welding interface, the maximum strength of 104 MPa was obtained in those welded joints [3].

Extensive studies have shown that high-entropy alloys have good weldability [21,22,23], which can significantly inhibit the formation of IMCs for dissimilar metal welding. A flux-cored welding wire using CoCrFeMoNiV complex concentrated alloy was investigated by Treutler et al. [24]. It was found that the signal FCC phase was discovered in WM in arc additive manufacturing. Kaviyarasan et al. [25] found that, when using the cable-like wires of Fe_0.2_Al_0.2_Ni_0.2_Cu_0.2_ and Fe_0.2_Al_0.2_Ni_0.2_Cu_0.2_Ti_0.2_, the presence of both FCC and BCC structures was observed in the welded joints, and the IMCs were inhibited, resulting in a 24.3% and 27.9% increase in joint strength. In the earlier study [26], the potential for joining Ti/steel bimetallic sheets via multi-principal powders of FeCoCrNiMn was indicated. However, owing to the melting of the base metal (BM) and dilution of the weld composition, it can significantly alter the high-entropy materials of the WM, which causes the formation of IMCs and degrades the mechanical properties of welded joints. Therefore, in this study, a multi-principal powder of CoCrNiMn is designed and utilized as a filler material in the welding of the TA1/Q345 bimetallic sheet. It is expected that the in situ formation of high-entropy alloys of Fe-Co-Cr-Ni-Mn-Ti would be achieved using the filler powders combined with the Fe and Ti elements from the melting of the BM so as to restrict the generation of FeTi and Fe_2_Ti and to attain the promising welded joints of the TA1/Q345 bimetallic sheet. Moreover, the microstructure of the welding interface and the composition of the WM will be studied. The mechanical properties and fracture mode of the welded joint have been evaluated.

## 2. Materials and Methods

**Figure 1:**
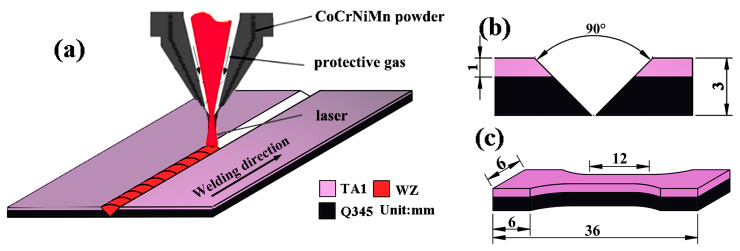
Experimental material diagram: (**a**) TA1/Q345 bimetallic sheet welded joint; (**b**) the parameter of groove; (**c**) tensile test specimen.

In this study, a hot-rolled TA1/Q345 bimetallic sheet was used as the BM. The TA1 layer had a thickness of 1 mm, while the Q345 layer had a thickness of 2 mm. The schematic diagram of the laser welding process of TA1/Q345 bimetallic sheets is manifested in Figure 1a. A 90° V-shaped groove was designed (see Figure 1b), which avoided the dilution of WM caused by excessive melting of the BM. Simultaneously, it can reduce the generation of Fe-Ti IMCs. Specifically, a mixture of powders consisting of CoCrNiMn was designed as the filler material. The original powders (Changsha Tianjiu Metal Material Co., Ltd., Changsha, China), including Co, Cr, Ni, and Mn, had a purity of 99.9 wt.% and a diameter ranging from approximately 24 to 75 μm, which were prepared with equal atomic ratio, that is, Co: Cr: Ni: Mn = 25 at.%: 25 at.%: 25 at.%: 25 at.%. The mass ratio of the multi-principal powders was Fe: Co: Cr: Ni: Mn = 26.24 wt.%: 23.15 wt.%: 26.14 wt.%: 24.46 wt.%. These powders were blended in a ball mill at 200 r/min for 120 min to reach a uniform composition.

An IPG YLS-4000-cl YAG fiber laser (USA) with a maximum power of 3000 W was utilized to conduct the welding process. The laser, shielding, and filler material were integrated into the nozzle and expelled synchronously. The argon gas, with a purity of 99.999%, was used as a shielding gas, and its flow rate was 15 L/min. An optimal set of welding parameters was used and displayed in Table 1. To avoid the influence of different welding parameters on the microstructure and mechanical properties of the WM, the same parameters were selected for each pass. After the welding process, the cross-section of the TA1/Q345 butt-welded joint, which was cut with an electric discharge machine, was processed for microstructural characterization. The metallographic specimen was then prepared through polishing and etching using aqua regia (HCl:HNO_3_ = 3:1) as the selected etchant. The microstructural characterization and metal compositions of the welded joint were investigated using a Zeiss Axio Vert.A1 metallographic optical microscope (OM) and a Hitachi SU8010 scanning electron microscope (SEM, Hitachi, Japan) mounted with Bruker Xflash 6I60 energy dispersive spectroscopy (EDS, Bruker, USA). In the SEM and EDS analyses, an accelerating voltage of 10 kV was adopted. For the tensile test, an AG-X 250 kN universal testing machine (Shimadzu, Japan) was employed. The dimensions of the tensile specimens are presented in Figure 1c. The tensile rate was established at 1 mm/min. Three dumbbell samples were chosen for each type of sample to ensure the accuracy of the tensile tests.

## 3. Results

### 3.1. Macro-Morphology

**Figure 2:**
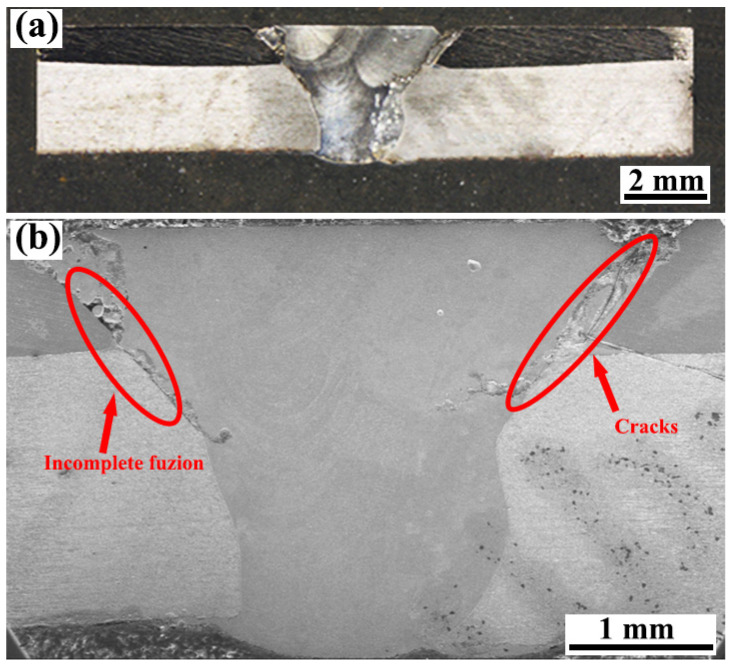
Cross-sectional morphology of welded joints: (**a**) photographed by stereomicroscope; (**b**) photographed by SEM.

Figure 2 illustrates the cross-sectional morphology of the joint of the TA1/Q345 layered bimetallic sheet after undergoing four-pass laser welding with CoCrNiMn filler powders. A clear outline of the molten pool is displayed for each welding pass. In Figure 2b, incomplete fusion and a crack of ~2.21 mm are observed at the interface of WM and TA1, with an angle of ~45° to the horizontal. It should be noted that those defects are located on the TA1 layer. The reasons are attributed to the significant differences in thermophysical and chemical properties between Ti and steel. It is well known that the thermal conductivity of TA1 is much lower than that of Q345, which results in higher thermal stresses during the welding process of the TA1 layer. In addition, the Ti/WM interface is prone to the generation of brittle IMCs owing to the fact that the diffusion rate of the Ti element is much faster than that of the Fe element [27]. The metallurgical bond between Q345 and the WM is satisfactory in comparison, as shown in Figure 2b. This finding has been confirmed by several previous studies [18,28]. The tensile test results of Li et al. [18] showed that the fracture occurred at the TC4/WM interface, while the steel/WM interface exhibited superior metallurgical bonding. Additionally, at the Q345/WM interface, the fracture appears as a dimple rather than a brittle cleavage pattern [28].

### 3.2. Interface Characteristics

**Figure 3:**
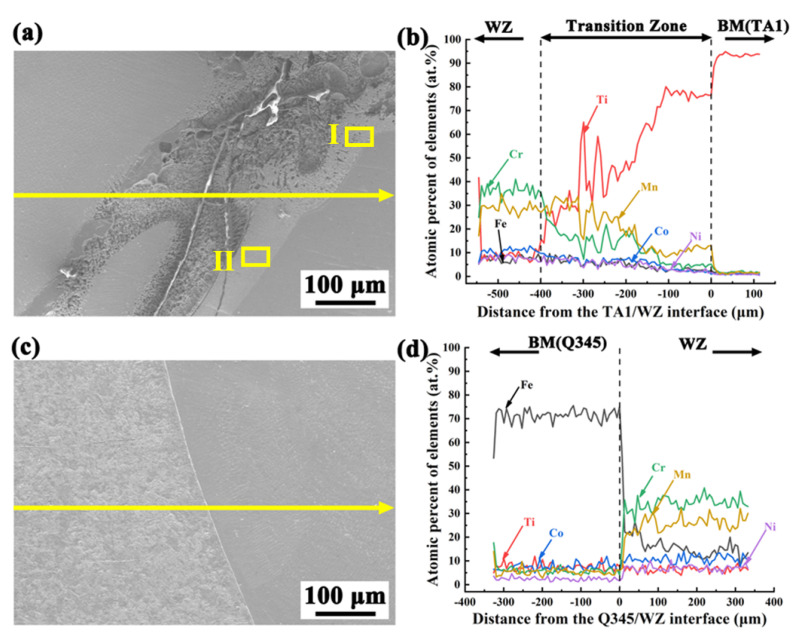
Distribution of elements at the interface: (**a**,**b**) the TA1/WM interface; (**c**,**d**) the Q345/WM interface.

Figure 3 displays the microstructure and distribution of elements at the interfaces between the BM and the WM. The results of the EDS line scanning in Figure 3b,d show the trend of the elements near the welding interfaces. Based on the element distribution, a distinct transition zone with a width of ~400 µm in the horizontal direction is observed on the TA1 layer. In this zone, the concentration of the Ti element is rapidly declining, from the BM towards the WM. However, the content of other elements, including Fe, Co, Cr, Ni, and Mn, is gradually increasing. The evident composition gradients at the transition zone indicate the diffusion and reaction of Ti, Mn, and Cr elements. It should be noted that similar transition zones have always been observed in previous studies. Previous experiments have reported that the transition zone was formed by the diffusion between the Ti layer and the WM [28]. During the laser welding of CP-Ti to AISI 304 stainless steel, Chattopadhyay et al. [29] identified a similar transition zone. The diffusion layer, composed of Ti and Cu elements, was predominant on the side of the Ti alloy. Compared to the Cr and Mn elements, the diffusion coefficient of Co and Ni elements is significantly lower, while the diffusion coefficient of Ti is higher [30]. Consequently, the content of Co and Ni elements is low in this transition zone (see Figure 3b).

**Figure 4:**
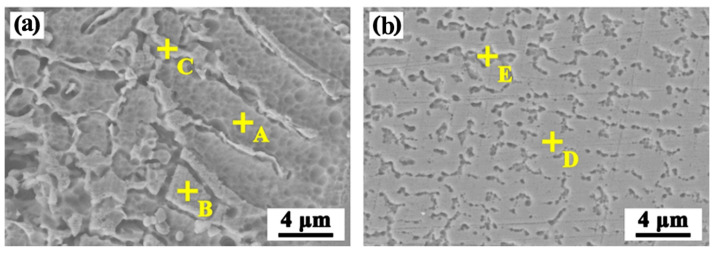
SEM morphologies of the TA1/WM interface in Figure 3a: (**a**) Region I; (**b**) Region II.

The microstructures of the transition zone in Figure 3 are carefully studied, and the SEM morphologies are illustrated in Figure 4. It means that the dendrite of different morphologies is displayed in Region I and Region II. The EDS point scanning results for typical regions are listed in Table 2. It is seen that the contents of Fe, Co, and Cr elements in those two regions are all less than 10 at.%. On the contrary, the content of the Ti element exceeds 50 at.%. Based on the information presented in Table 2 and the phase diagram of ternary alloys (Ti-Ni-Mn) [31], the dendrite structures of Region I and Region II are mainly composed of a Ti-rich phase, which may be β-Ti.

A clear fusion line between the Q345 steel and WM, with no transition zone, is observed in Figure 3c. Epitaxial solidification is observed as the grains in the weld edge grow attached to the Q345 boundary. The grains display directionality towards the WM and feature characteristic columnar crystal morphology [32]. According to the results from Sokkalingama et al. [33], the growth of columnar crystals is directly influenced by the rate of heating and cooling. During the welding process of the CoCuFeMnNi high-entropy alloy, Fiocchi et al. [34] have noted significant dendritic epitaxial growth on the steel side. Moreover, enormous variations in elemental content appear around the interface of Q345/WM, as shown by the EDS line scan results in Figure 3d. The content of each element in the WM is less than 50 at.%. There is a significant decrease in Fe elemental content compared to the Q345 BM, while the content of Mn and Cr elements is dramatically increased, accounting for ~70 at.%. Additionally, it should be noted that the filler material used in this study does not contain any Fe element, but a content of 10~20 at.% for the content of Fe is found in the WM. It means that the Fe element has been introduced into the weld metals as a result of the melting of the BM, and the multi-principal element alloy of Fe-Co-Cr-Ni-Mn-Ti has been successfully achieved in the WM.

### 3.3. Microstructure in the WM

**Figure 5:**
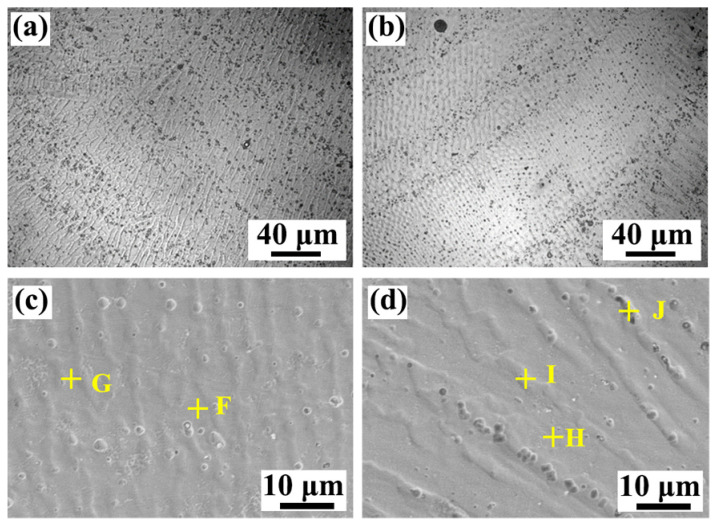
Morphology of the weld center: (**a**,**b**) OM images; (**c**,**d**) SEM images.

Figure 5a,b illustrate the microstructure in the weld center. It is observed that refined dendrites are observed in the weld center. It means that the grain size is too small, and the grain boundaries cannot be clearly observed in an OM image. Some black-dotted phases are found at the grain boundary. SEM was used to observe the microstructures more clearly. In Figure 5c,d, it is evident that the black dotted phase is predominantly an enhancement of grain boundaries. In the previous experiments, the microstructure in the weld was dominated by coarse dendritic morphology and a large amount of a-Fe + TiFe_2_ eutectic and TiFe_2_ + (a-Fe + TiFe_2_) hypereutectic structures [26,28]. The microstructure of the WM in this experiment is completely different from the microstructure morphologies that appeared in previous experiments, which are mainly fine columnar crystals with a width of about ~5 μm in Figure 5c and ~10 μm in Figure 5d, respectively. The reason is mainly related to the difference in chemical composition. Based on the results in Table 3, the major elements of black phases are Cr, Ni, and Mn elements, constituting contents beyond 20 at.%. The concentrations of Fe, Co, Cr, Ni, and Mn, as observed, range from 5 at.% to 35 at.%. It should be stated that the results are well in accordance with the composition of the high-entropy alloy [35].

An interesting observation is that, according to the results in Table 3, the concentration of the Ti element is very low in the weld center, with a concentration not exceeding 1.0 at.%. In fact, similar trends can be observed in Figure 3. The reduction in Ti content within the WM, in comparison to the transition zone located on the Ti layer, is readily apparent (see Figure 3b). In addition, the Ti element is almost undetectable in the WM on the Q345 steel layer (see Figure 3d). It should be noted that the experiment results are significantly different from some previous studies [26]. Liu et al. [26] have reported that a high content of the Ti element (8.0~27.3 at.%) was detected in the weld center for the welded joints of TA1/Q345 bimetallic sheets with multi-principal filler powders of FeCoCrNiMn. Moreover, a content of 16.5~29.9 at.% for the Ti element was found in the weld center by applying a Ni-Co-Al multi-principal wire [28]. The limited presence of the Ti element in the weld center can be attributed to two underlying factors. First, a high-angle, 90° V-shaped groove has been used in the present study, which is much larger than that in previous studies (30~45°). The presence of this phenomenon leads to a decrease in the concentration of the Ti element within the WM. Second, four weld passes are used to completely fill the V-shaped groove. Therefore, the main elements in the WM are those elements from the filler material of CoCrNiMn.

### 3.4. Tensile Strength and Fracture Behavior

**Figure 6:**
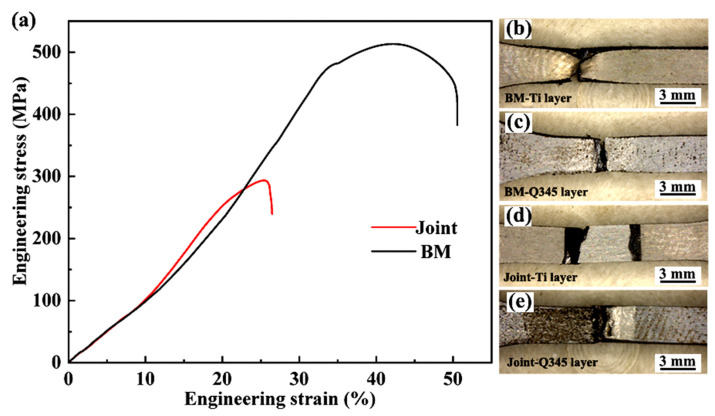
Tensile properties and fracture behavior of the BM and welded joints: (**a**) the stress–strain curves; (**b**–**e**) the fracture positions.

The representative tensile curves for the welded joint and TA1/Q345 bimetallic sheet are illustrated in Figure 6a. It is seen that the measured value for the tensile strength of the BM is ~488 ± 18 MPa. Notably, a necking phenomenon is observed on the TA1 layer for the fractured BM sample, as shown in Figure 6b. However, similar necking observations are hardly found on the Q345 layer, as represented in Figure 6c. Figure 6a indicates that the tensile strength of the welded joint is ~293 ± 12 MPa. The joint efficiency is ~60%. It is noteworthy that the strength value in this investigation surpasses the values reported in numerous prior studies [26,28]. As reported in a recent study, the welded joint of TA1/Q345 bimetallic sheets applying the Ni-Co-Al multi-principal wire has achieved a tensile strength value of 123 MPa [28] as well as a value of 117 MPa in the welded joint applying FeCoCrNiMn powders as filler material [26]. The main reason for the increase in the strength value of the welded joint may be attributed to the decrease in Ti content in the WM. Nonetheless, the strength value is lower compared to the experimental results of Zhang et al. [32]. It may have a close relationship with the presence of large welding defects in this study.

**Figure 7:**
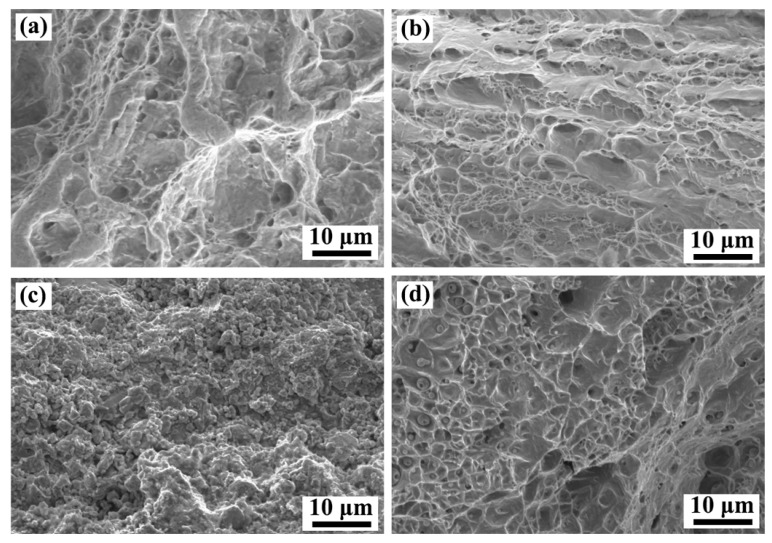
Fracture morphologies: (**a**) BM on the TA1 layer; (**b**) BM on the Q345 layer; (**c**) Joint on the TA1 layer; (**d**) Joint on the Q345 layer.

The fracture position of the welded joint is displayed in Figure 6d–e. It is seen that the fracture is located in the weld toes along the incomplete fusion side. No significant necking is observed in the fractured sample. To better understand fracture behavior, fracture surfaces were studied. The fracture morphologies of the BM and joint are displayed in Figure 7. Numerous dimples are visible on the fracture surface of the BM, indicating a ductile fracture pattern. In the case of the welded joint, large differences in the morphologies can be observed on the TA1 and Q345 layers. For the TA1 layer, the fracture surface displays a granular morphology, as shown in Figure 7c. It means that a relatively weak metallurgical connection may be present between the TA1 BM and the WM. Ning et al. [9] have discovered a similar morphology in the Cu filler layer during the welding process of Ti/steel composite plates, and the granular morphology on the fracture surface was established as a Cu solid solution and FeTi. It further confirms that the welding interface produces a brittle fracture surface on the TA1 layer. For the Q345 layer, Figure 7d depicts many dimples on the fracture surface. Spherical particles are seen at the bottom of the dimples. It proves the good plasticity of the welded joint on the Q345 layer. Based on previous studies [36], spherical particles are also visible on the fracture surface of the welded joints of high-entropy alloys and 316L stainless steel. According to the EDS analysis, spherical particles should be Cr-rich and Mn-rich oxides and carbides [36].

## 4. Discussion

### 4.1. Weld Formation

Figure 2 indicates that welding defects, including incomplete fusion and cracks, are found at the interface of WM and TA1. Based on the position of the fracture occurring in the welded joint displayed in Figure 6d,e, the welding defects have a significant effect on the fracture behavior and tensile strength. Uncovering the mechanism of defect formation is essential for preventing defects and improving the tensile strength of the welded joint. As is known, the welding defects may be caused by the presence of oil stains on two sides of the groove, the angle of the groove, or inappropriate welding parameters. Yu et al. [37] have suggested that, owing to the varying distance of the laser beam, the heat input from the laser beam into the two sides of the groove is not uniform. It can result in the presence of inadequate fusion. Li et al. [38] have proposed that a mismatched welding parameter can change the fluidity of the liquid metal in the weld pool, leading to the formation of incomplete fusion in the welded joints. Based on previous studies [38,39], it can be speculated that the presence of inadequate fusion in this study has a close relationship with the mismatched groove angle, weld pass, and welding parameters.

Moreover, cracks are found in the transition zone on the TA1 layer. Two primary reasons may be attributed to those cracks. First, the metallurgical reaction produces substantial hard and brittle IMCs in the transition zone, severely weakening the plasticity of the transition zone [40]. Second, a high thermal stress should be formed at the interface of WM and TA1 during the heating and cooling of the welding process [9]. The impact of stress on crack generation can be assessed with a rough calculation with the equation σ = E × α × ΔT, where α, ΔT, and E are the coefficients of thermal expansion (CTE), the temperature difference, and the Young’s modulus, respectively. Assuming a constant and the same differential temperature (ΔT_TA1_ = ΔT_Q345_) during welding for both TA1 and Q345. The Young’s modulus and CTE values of Ti and Fe are 8.6 × 10^−6^ K^−1^ and 11.0 × 10^−6^ K^−1^, respectively [11]. The Young’s moduli of Ti and Fe are 104.2 GPa and 201.8 GPa, respectively [41]. Therefore, the thermal stress in the Q345 layer is ~2.5 times greater than that in the TA1 layer. As a result of the combination of thermal stress and IMCs, cracks are generated in the transition zone on the TA1 layer.

Based on the experimental results, some welding defects, such as incomplete fusion and cracks, are observed in this transition zone between the WM and BM on the TA1 layer. To avoid those welding defects, first, the laser scanning path and welding parameters could be further optimized. Second, the shape and angle of the welding groove can be adjusted. Third, advanced fusion welding methods, such as cold metal transfer (CMT), may be an ideal choice to improve the welding formation.

### 4.2. High-Entropy Structures and Tensile Strength

As is known, based on the definition of high-entropy alloys, the content of each main element in the alloys is in the range of 5~35 at.% [42]. Moreover, the entropy of mixing (ΔS_mix_) should be higher than 1.5 R. From the Table 3 results, the elemental contents of Fe, Co, Cr, Ni, and Mn, excluding the Ti element, are located in the range of 5~35 at.%. It is well in accordance with the definition of a high-entropy alloy from an elemental perspective. To further analyze whether the WM has achieved a high-entropy alloy structure, some thermodynamic parameters, such as the ΔS_mix_, the enthalpy of mixing (ΔH_mix_), the atomic size difference (δ), the Ω parameter, the electronegativity difference (∆χ_Allen_), the valence electron concentration (VEC), and the average value of the d-orbital energy level ($\bar{Md}$), were calculated, and the results are presented in Table 4. It is seen that the values of ΔS_mix_ for all the measured points are higher than 1.5 R (12.47 J mol^−1^ K^−1^). It means that it can satisfy the conditions for the formation of high-entropy alloys in the WM, which is in agreement with the results of previous studies [26].

Moreover, many previous studies have reported that a single high-entropy solid solution phase forms when 12 ≤ ΔS_mix_ ≤ 17.5 J·K^−1^·mol^−1^, −15 ≤ ΔH_mix_ ≤ 15 kJ·mol^−1^, δ ≤ 6.6%, and Ω ≥ 1.1 [42,43,44,45]. Poletti et al. [44] have noted that solid solutions are formed if δ < 6% and ∆χ_Allen_ < 6%. No TCP phases form due to $\bar{Md}$ < ~0.95 [43]. Kumar et al. [45] found that a solid solution phase of the FCC structure is obtained when VEC ≥ 7.95, and a single solid solution phase of the BCC structure is more stable when VEC < 7.2. FCC, BCC, and FCC + BCC solid-solution phases are formed when 7.2 ≤ VEC < 7.95. According to the results in Table 4, it is meant that solid solutions of all three combinations should be produced in the WM rather than the IMCs.

Based on the tensile results of the welded joint in this work, the tensile strength of the welded joint in the present study (~293 MPa) is much higher than that of those welded joints employing multi-principal filler metals (117~123 MPa). The reason is mainly ascribed to the decrease in Ti elements in the WM in the present study. Liu et al. [28] have suggested that excessive Ti elements can lead to the generation of Fe-Ti IMCs in the WM. In addition, owing to its comparatively considerable atomic radius in relation to other elements, the solute atom of Ti can induce substantial lattice distortion, which can lead to a significant reduction in the overall tensile strength of the welded joint. Nonetheless, the tensile strength is lower than some previous studies about the welded joints of Ti/steel bimetallic sheets. Zhang et al. [10] found that joint strength can reach ~467 MPa due to the presence of an Fe-based solid solution in the WM. Moreover, Zhang et al. [32] found that a joint strength of ~317.6 MPa can be achieved for the fusion brazing of TA2/Q235 bimetallic sheets. It is noteworthy that the presence of solid solutions, rather than IMCs, in the WM can significantly improve the tensile strength of the welded joints of Ti/steel bimetallic sheets.

## 5. Conclusions

Based on the research presented in this paper, the following conclusions have been drawn:

(1) Multi-principal powders of CoCrNiMn can be used as filler material for the joining of the TA1/Q345 bimetallic sheet. High-entropy alloy structures, with the content of Fe, Co, Cr, Ni, and Mn elements in the range of 5~35 at.%, as well as a high ΔS_mix_ value were attained in the weld metal.
(2) A transition zone with a width of ~400 µm was presented between the weld metal and base metal on the TA1 layer. Some welding defects, such as incomplete fusion and cracks, were observed in this transition zone.
(3) A tensile strength of 293 MPa, accounting for 60% of the strength exhibited by the base metal, was achieved in the joining of the TA1/Q345 bimetallic sheet. Dimples were observed at the fracture surface of the welded joint on the Q345 layer.

## Figures and Tables

**Table 1:** Welding parameters.

Laser Power	Groove Type	Root Gap	Filler Powders	Spot Diameter	Welding Speed	Defocus Distance	Powder Feed Rate	Number of Weld Passes
900 W	90° V-shaped	0	CoCrNiMn (24–75 μm)	2 mm	120 mm/min	1 mm	14 g/min	4 passes

**Table 2:** EDS analysis of some feature points in the WM/TA1 interface.

A	B	C	D	E	Test Points	A	B	C	D	E
at.%						wt.%				
2.56	5.54	2.21	2.30	2.75	Fe	2.82	6.12	2.39	2.52	3.08
1.68	1.68	3.36	8.70	5.39	Co	1.95	1.96	3.83	10.06	6.38
-	1.72	2.53	-	-	Cr	-	1.77	2.55	-	-
7.13	11.89	14.84	14.52	11.91	Ni	8.25	13.81	16.86	16.72	14.03
24.34	9.69	21.57	5.14	-	Mn	26.35	10.53	22.94	5.54	-
64.28	69.48	55.49	69.34	79.95	Ti	60.64	65.81	51.43	65.15	76.51
β-Ti					Potential phase	β-Ti				

**Table 3:** EDS analysis of some feature points in the WM.

F	G	H	I	J	Test Points	F	G	H	I	J
at.%						wt.%				
11.30	17.01	5.07	7.81	18.94	Fe	11.34	17.09	5.09	7.86	19.16
9.36	13.29	12.85	15.45	7.70	Co	9.91	14.09	13.61	16.42	19.16
21.86	28.13	27.74	33.58	27.34	Cr	20.42	26.32	25.92	31.48	25.75
24.32	21.84	25.91	22.42	15.93	Ni	25.64	23.07	27.32	23.73	16.94
33.01	19.67	28.43	20.45	29.98	Mn	32.58	19.39	28.06	20.26	29.83
0.14	0.05	-	0.29	0.12	Ti	0.12	0.04	-	0.25	0.10

**Table 4:** Calculation results of thermodynamic parameters.

Test Point	ΔH_mix_	ΔS_mix_	δ (%)	$\bar{Md}$	Ω	∆χ_Allen_ (%)	VEC
F	−4.88	12.63	3.83	0.913	4.58	4.71	7.81
G	−4.38	13.15	3.26	0.916	5.50	4.94	7.81
H	−5.22	12.29	3.62	0.918	4.24	5.03	7.81
I	−4.98	12.72	3.35	0.933	4.73	5.33	7.72
G	−3.47	12.71	3.75	0.938	6.62	4.62	7.54

## Data Availability

Research data are not shared.
